# Nanostructured ZnO thin film to enhance gutta-percha’s adhesion to endodontic sealers

**DOI:** 10.1186/s12903-024-04496-z

**Published:** 2024-06-29

**Authors:** Inês Ferreira, Cláudia Lopes, Armando Ferreira, Ana Cristina Braga, Filipe Vaz, Irene Pina-Vaz, Benjamin Martin-Biedma

**Affiliations:** 1 School of Medicine and Dentistry, University of Santiago de Compostela, Santiago de Compostela, Spain; 2 CINTESIS, R&D Unit, Faculty of Medicine, University of Porto, Porto, Portugal; 3 Faculty of Dental Medicine, University of Porto, Porto, Portugal; 4 Physics Centre of Minho and Porto Universities (CF-UM-UP), University of Minho, Braga, Portugal; 5 LaPMET—Laboratory of Physics for Materials and Emergent Technologies, University of Minho, Braga, Portugal; 6 Department of Production and Systems, ALGORITMI Center, University of Minho, Braga, Portugal; 7 Oral Sciences Research Group, Endodontics and Restorative Dentistry Unit, School of Medicine and Dentistry, University of Santiago de Compostela, Health Research Institute of Santiago (IDIS), Santiago de Compostela, Spain

**Keywords:** Adhesiveness, Calcium silicate-based sealer, Epoxy resin-based root canal sealer, Gutta-percha, Plasma treatment, ZnO thin film

## Abstract

**Background:**

Gutta-percha (GP) combined with an endodontic sealer is still the core material most widely used for tridimensional obturation. The sealer acts as a bonding agent between the GP and the root dentinal walls. However, one of the main drawbacks of GP core material is the lack of adhesiveness to the sealer. ZnO thin films have many remarkable features due to their considerable bond strength, good optical quality, and excellent piezoelectric, antibacterial, and antifungal properties, offering many potential applications in various fields. This study aimed to explore the influence of GP surface’s functionalization with a nanostructured ZnO thin film on its adhesiveness to endodontic sealers.

**Methods:**

Conventional GP samples were divided randomly into three groups: (a) Untreated GP (control); (b) GP treated with argon plasma (PT); (c) Functionalized GP (PT followed by ZnO thin film deposition). GP’s surface functionalization encompassed a multi-step process. First, a low-pressure argon PT was applied to modify the GP surface, followed by a ZnO thin film deposition via magnetron sputtering. The surface morphology was assessed using SEM and water contact angle analysis. Further comprehensive testing included tensile bond strength assessment evaluating Endoresin and AH Plus Bioceramic sealers’ adhesion to GP. ANOVA procedures were used for data statistical analysis.

**Results:**

The ZnO thin film reproduced the underlying surface topography produced by PT. ZnO thin film deposition decreased the water contact angle compared to the control (*p* < 0.001). Endoresin showed a statistically higher mean bond strength value than AH Plus Bioceramic (*p* < 0.001). There was a statistically significant difference between the control and the ZnO-functionalized GP (*p* = 0.006), with the latter presenting the highest mean bond strength value.

**Conclusions:**

The deposition of a nanostructured ZnO thin film on GP surface induced a shift towards hydrophilicity and an increased GP’s adhesion to Endoresin and AH Bioceramic sealers.

## Introduction

The success of endodontic treatment depends on canal shaping, cleaning, disinfection protocols, and the hermetic filling of the root canal system. The main goal of root canal obturation is to prevent coronal and apical leakage and entomb the remaining bacteria that may persist after treatment [1]. Gutta-percha (GP) combined with an endodontic sealer is still the core material most widely used for tridimensional obturation. The sealer acts as a bonding agent between the GP and the root dentinal walls. Commercially available endodontic sealers vary in composition, leading to different interactions with dentin and GP [2–4]. Epoxy resin-based sealers are widely recommended for root canal filling due to their good physical properties [5]. Calcium silicate-based sealers (CSS) have gained prominence due to their high biocompatibility, bioactive feature, and antimicrobial action [6]. The bond strength of root canal sealers has been tested mainly to the dentinal walls, whilst there is still a lack of knowledge regarding their adhesion ability to core-filling materials, which might influence sealing ability and filling resistance to dislodgement [4, 7]. In this study, two sealers were tested, an epoxy resin-based sealer – Endoresin, and a calcium silicate-based sealer – AH Plus Bioceramic. AH Plus Bioceramic is a recent premixed calcium silicate-based sealer comprising zirconium dioxide, tricalcium silicate, dimethyl sulfoxide, lithium carbonate, and a thickening agent [8]. While it exhibits favorable physical properties and antibacterial activity due to its high pH, its solubility may impact the obturation quality [9]. Recent studies have highlighted its cytocompatibility and bioactive potential, surpassing the epoxy resin-based AH Plus and rivaling EndoSequence BC Sealer [8]. Epoxy resin-based sealers, like Endoresin, has been used as the gold standard material due their physicochemical properties [10]. There is a lack of knowledge regarding the performance of AH Plus Bioceramic and Endoresin in terms of bond strength to GP.

Until now, none have supplanted GP, which continue to be universally accepted as the ‘gold standard’ core filling in root canal treatment [11]. The commercially denominated GP is a polymer that contains approximately 20% of GP (matrix), 66% of zinc oxide (filler), 11% of heavy metal sulfates (radiopacifier) and 3% of waxes and/or resins (plasticizer) [12]. One of the main drawbacks of GP core material is the lack of adhesiveness to the sealer. Its hydrophobic nature tends to pull the sealer away during setting [13]. In the last few years, studies have been developed to find a way to improve the characteristics of GP [11, 14–18]. The concept of coating the GP by methacrylate resin, glass ionomer, apatite calcium phosphate and nanoparticles (chitosan, silver and bioceramic) has emerged, to enhance its antimicrobial characteristic and adhesion ability [11]. However, there is still a gap in achieving a better long-term fluid-tight seal between the GP core and the sealer [16].

Zinc oxide (ZnO) constitutes a significant portion of GP’s elemental composition [19]. Classified as safe by the US Food and Drug Administration, ZnO is a low-cost material that is easy to process, abundant in nature, biosafe, biocompatible, and non-toxic, presenting antimicrobial activity [14, 20]. At the nanometric scale, ZnO thin films have many remarkable features due to their considerable bond strength, good optical quality, and excellent piezoelectric, antibacterial, and antifungal properties, offering many potential applications in various fields [21–23]. A previous study proposed a novel approach to increase the antibiofilm efficacy of GP, modifying its surface by using Argon (Ar) plasma treatment (PT), followed by the deposition of a ZnO thin film [14]. PT has several applications, and one of the most used is directly related to the modification and functionalization of the materials’ surfaces [15, 24, 25], in a controlled, reproducible, and homogeneous way without changing the main properties of the bulk material [25–28].

The interaction of plasma with surfaces is greatly affected by the energy input, pressure, working gas composition and the nature of the substrate [26, 31]. Depending on these factors, a diversity of chemical-reaction based interactions with surface materials can be enhanced or even enabled by plasma application, including cleaning, activation, etching or ablation, and film thin deposition (coating) [25].

Although the functionalized GP with ZnO thin film has shown promising results in terms of biocompatibility and antibacterial properties [14], to our knowledge, no studies have investigated its performance concerning sealers’ adhesion. Therefore, the present investigation focused on the influence of GP surface’s functionalization with a nanostructured ZnO thin film deposition on the adhesion to an epoxy resin-based and a calcium silicate-based endodontic sealer. The null hypothesis was that GP surface’s functionalization will not have impact on sealer adhesion.

## Materials and methods

### Materials used

#### Gutta-Percha (GP)

Round disks of GP samples (10-mm diameter and 2-mm thickness) were produced from GP pellets (Gutta-percha Bal Plus Pellets; Meta Biomed Co, Ltd; Korea) by creating appropriate molds and then plasticizing GP in a laboratory dry-heating oven at 80 °C, followed by a cooling process at room temperature. A standardized metallographic procedure was employed to produce samples with similar surface roughness, for that the specimens were polished with coarse silicon carbide abrasive papers (180 to 600 grit). The GP round disks were used to surface morphology and contact angle analysis. Additionally, GP pellets submitted to the same standardized metallographic procedure, on its two flat sides, were used for the tensile bond strength testing with the sealers.

#### Sealers

- AH Plus Bioceramic (calcium silicate-based sealer; manufactured by Maruchi; distributed by Dentsply DeTrey GmbH).
- Endoresin (epoxy resin-based sealer; manufactured by Meta Biomed. Ltd, Chungcheongbuk-do, Korea; distributed by Galician Endodontics Company S.L., Lugo, Spain).

### Functionalization of GP

#### Plasma treatment (PT)

GP was submitted to PT in an Ar atmosphere using a low-pressure plasma cleaner (Plasma System Zepto; Diener electronic) powered at 50 W for 60 s. The working pressure never exceeded 80 Pa, while the base was kept at 20 Pa for all treatments. These parameters were applied based on a previous study [27].

### Deposition of ZnO thin film

The ZnO thin film was deposited onto GP by reactive magnetron sputtering at a working pressure of 5 × 10^− 1^ Pa, while keeping the flow of Ar (30 sccm) and O_2_ (20 sccm) constant using a reactive chamber with a volume of 50 dm^3^. A metallic zinc target with 99.96% purity was used for the depositions, with a 50.6-mm diameter and 6-mm thickness. The base pressure was kept lower than 4 × 10^− 4^ Pa, and the Zn target was connected to a DC source, setting the target potential at -378 V.

**Fig. 1:**
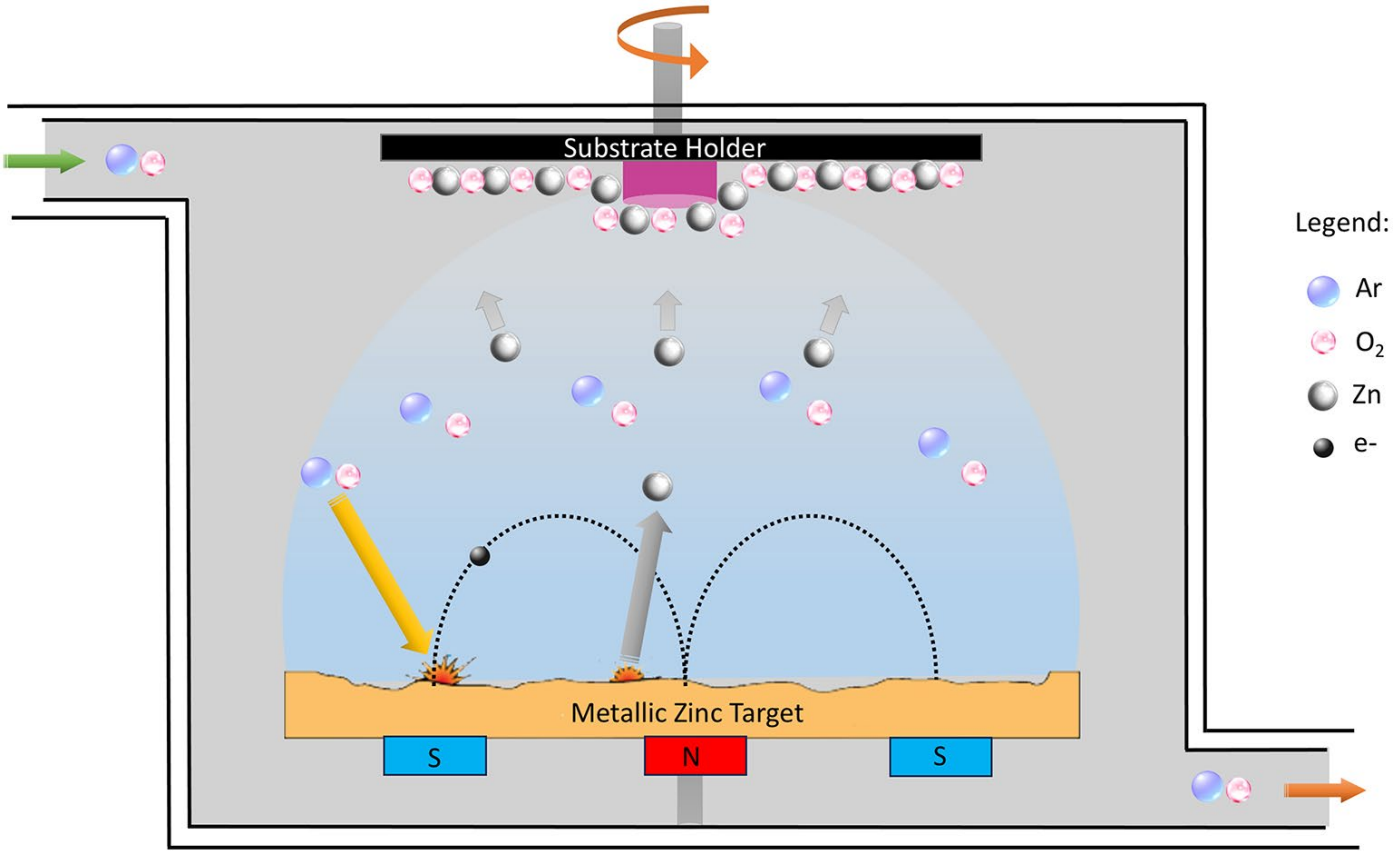
Schematic illustration of physical vapor deposition with reactive magnetron sputtering used in the production of ZnO thin films

The process of physical vapor deposition using reactive magnetron sputtering is depicted in Fig. 1. In this process, a target material, typically a metal or compound, is bombarded with Ar^+^ ions inside a vacuum chamber. The collision of Ar^+^ ions with the target result in a momentum and energy transference. As a consequence, the atoms of the target material are displaced from their original locations and ejected into the surrounding vacuum interacting with the O_2_ reactive gas, and then deposited on the GP substrate.

### Surface morphology analysis

The surface morphology of untreated (control) and treated GP surfaces (PT and PT followed by ZnO thin film deposition) was evaluated qualitatively by scanning electron microscopy (SEM) (*n* = 3). GP round disks surfaces were sputter-coated with a thin conductive film of Au-Pd alloy and then analyzed using an FEI Quanta 400 FEG/ESEM microscope in a vacuum at 15-kV accelerating voltage. The images obtained were qualitatively evaluated regarding the presence of morphological changes.

### Contact angle analysis

The G*Power v3.1.9.6 program was used to determine an a priori sample size. The procedure used was an analysis of variance (ANOVA) with repeated measures, within factors, using an alpha-type error of 0.05 with a power (1-ß) of 0.90, with an effect size of 0.4. Fifteen specimens per group were established as the ideal size. GP round disks were divided randomly into three groups: (a) Untreated GP (control); (b) GP treated with argon plasma (PT); (c) Functionalized GP (PT followed by ZnO thin film deposition). The samples were randomly allocated using an online computer-generated number (www.randomizer.org).

The contact angle was measured by the sessile drop technique. One drop per each sample of 0.5 mL of distilled water was dispensed on the surface of the GP round disks using a micro-syringe, and images were captured at room temperature using the optical contact angle equipment (OCA 20; DataPhysics Instruments GmbH, Filderstadt, Germany). After the water was applied on the GP surface for 5 s, the angle of contact was recorded [28]. Contact angle measures (*n* = 15) were conducted on the untreated (control), treated with Ar plasma (PT), and functionalized (PT + ZnO thin film) GP surfaces.

### Tensile bond strength

The G*Power v3.1.9.6 program was used to determine an a priori sample size. The procedure used was ANOVA with fixed effects, main effects, and interactions, using an alpha-type error of 0.05 with a power (1-ß) of 0.80 and six groups, with an effect size of 0.4. Ten specimens (each specimen refers to 2 pellets) per group were established as the ideal size. Samples were divided randomly into two groups, according to the sealer: Endoresin and AH Plus Bioceramic. Each group was subdivided into three sub-groups: (a) Untreated GP (control); (b) GP treated with argon plasma (PT); (c) Functionalized GP (PT followed by ZnO thin film deposition). The samples were randomly allocated using an online computer-generated number (www.randomizer.org).

**Fig. 2:**
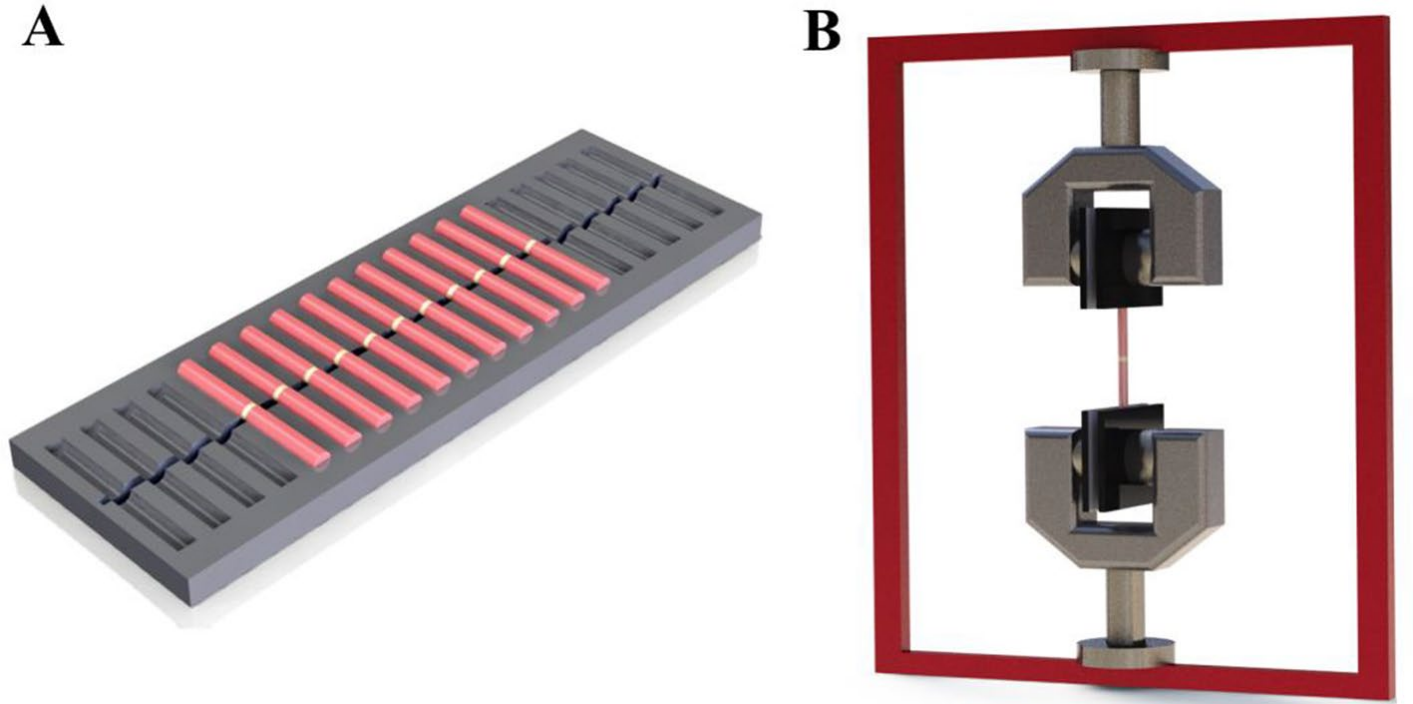
Schematic representation of the custom-designed apparatus for bonding the gutta-percha surface and the sealer (**A**), and the tensile bond strength test apparatus (**B**)

A 0.01-mL droplet of each sealer tested was precisely dispensed onto the central region of a flat-surfaced GP pellet using an automatic micropipette. Subsequently, an identical pellet was carefully aligned and affixed against the initial one (*n* = 10; 1 sample refers to 2 pellets), in a specially developed mold Fig. 2A. Any excess extruded material was meticulously removed with a dental microbrush applicator tip. The prepared samples were then stored at 37 °C, in contact with gauze moistened with a phosphate-buffered saline solution (pH 7.2) for 7 days. Any nonstandard sample was promptly replaced. The bond strength between the GP surface and the sealer was evaluated using a custom-designed apparatus, illustrated in Fig. 2B. After ensuring the samples’ stabilization, the moving part of the container was attached to the tensile machine. The measurements were conducted while subjecting the GP pellets to a tensile force, using a universal testing machine from Shimadzu (model AG-IS) equipped with a 50-N load cell at a 0.5-mm/min speed. The tensile bond strength test was performed in a random order by an operator blinded to the specific sealer under test. Bond strength was determined by a real-time computer software program that plotted a load/time curve during the test. The tensile force required to separate the GP pellets was recorded in Newtons (N), and the tensile bond strength in Mega Pascals (MPa), considering the GP pellets sectional area.

### Statistical analysis

The statistical analysis was performed using the IBM SPSS Statistic 28.0. software (SPSS Inc, Chicago, Illinois, EUA). The significance level was set at 5% (*p* < 0.05). The data were verified with the Kolmogorov-Smirnov test for the normality of the distribution and the Levene test for the homogeneity of variances. Water contact angle results were evaluated using ANOVA repeated measures (3 levels: control, PT, and PT + ZnO). Tensile bond strength results were analyzed with a two-way ANOVA followed by Bonferroni post-hoc tests. All the conditions for applying the ANOVA procedure were evaluated based on the residuals (normality, zero mean, homogeneity of variance, and independence).

## Results

### Surface morphology analysis

**Fig. 3:**
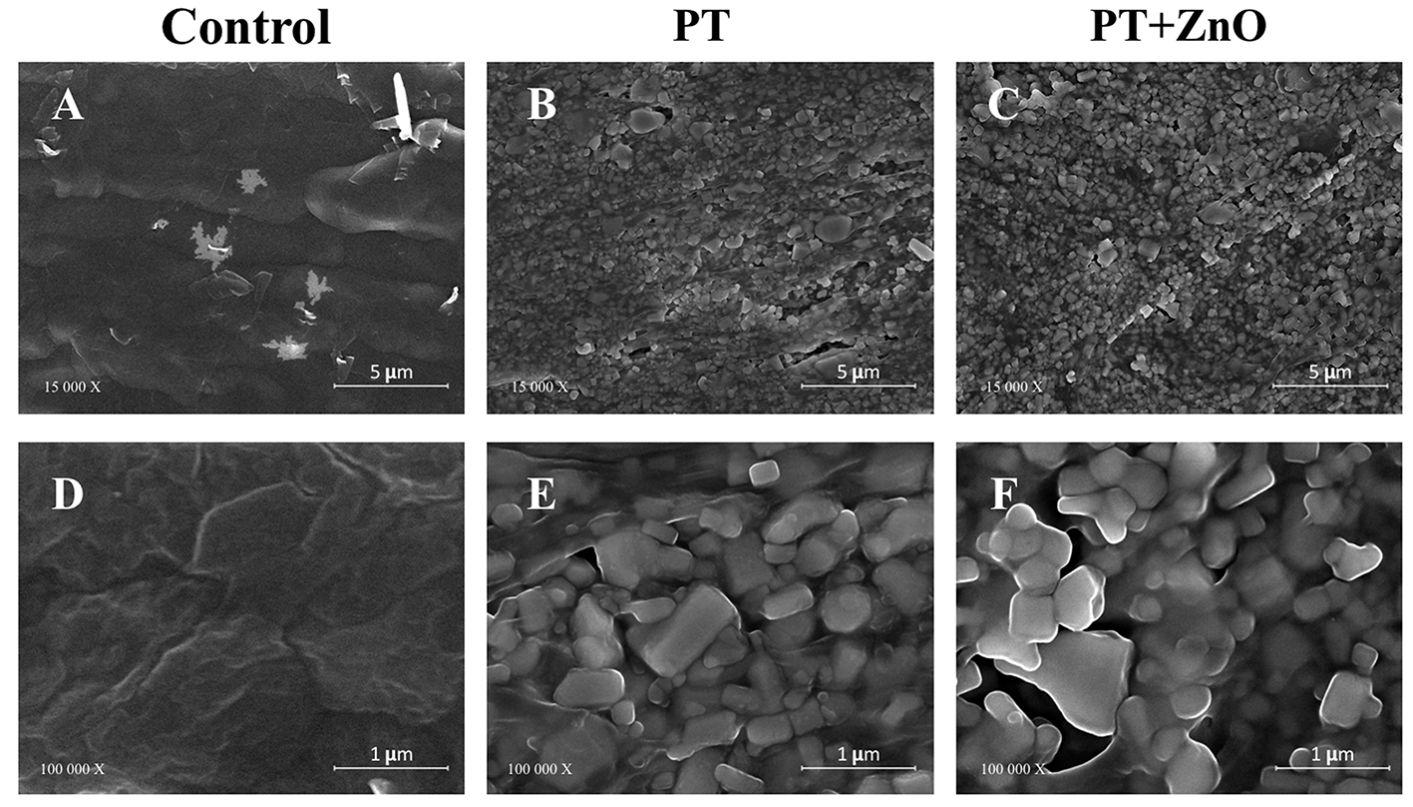
Representative scanning electron microscopy images of untreated (control; **A** and **D**), treated with argon plasma (plasma treatment - PT; **B** and **E**), and functionalized gutta-percha surfaces (plasma treatment following ZnO thin film deposition – PT + ZnO; **C** and **F**)

Figure 3 shows the SEM surface morphology analysis of the distinct GP surfaces: untreated (control), treated with Ar plasma (PT), and functionalized (PT + ZnO thin film). Untreated GP (control) showed ZnO crystals encrusted on the GP matrix, covered by an organic layer constituted by wax/resin components (Fig. 3. A and D). PT with Ar caused the removal of the wax/resin surface layer, exposing the ZnO crystals embedded in the GP matrix and uncovering the surface porosity promoted by the ZnO grains boundaries (Fig. 3. B and E). The GP samples submitted to PT + ZnO increased the surface area to be covered with ZnO, benefiting from its additional properties (Fig. 3. C and F).

### Contact angle analysis

**Fig. 4:**
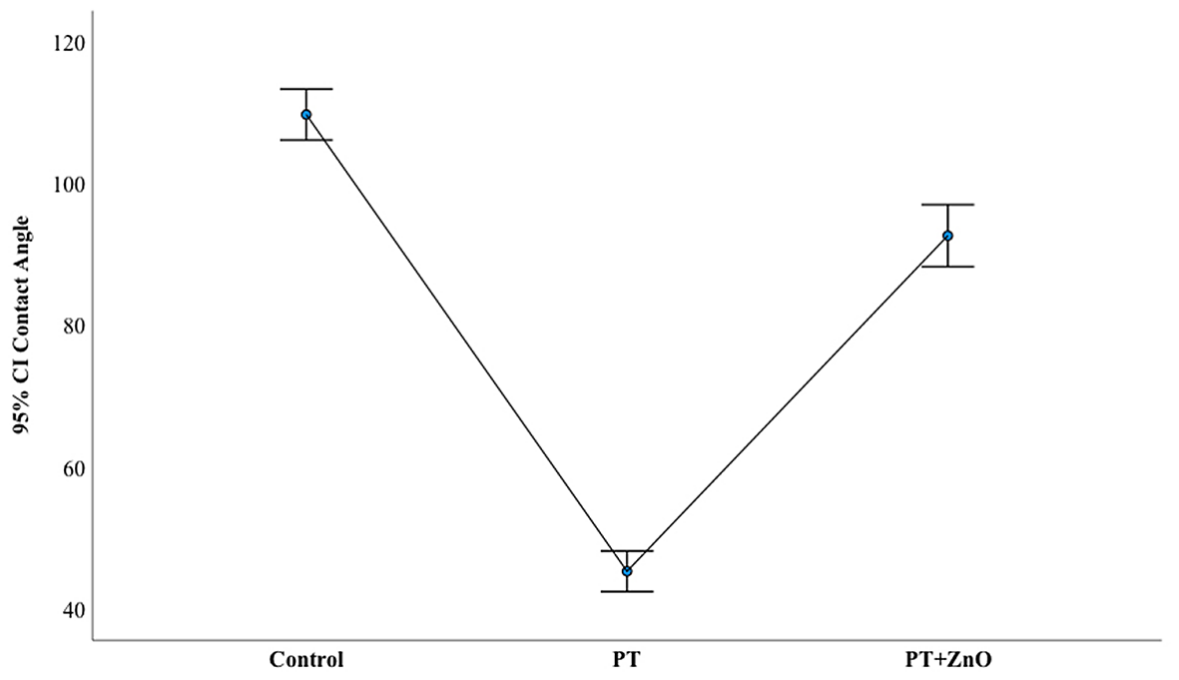
Mean distribution water contact angles in the distinct gutta-percha surfaces (*n* = 15): untreated (control), treated with argon plasma (PT), and functionalized gutta-percha surfaces (plasma treatment followed by ZnO thin film deposition – PT + ZnO)

Figure 4 illustrates water contact angles evolution of GP surfaces after plasma treatment (PT) and functionalization (PT + ZnO) from their pristine form (control). The water contact angles were significantly reduced after PT (*p* < 0.001) and functionalization with PT + ZnO (*p* < 0.001), comparing to the control. The difference between the mean water contact angle of PT and PT + ZnO was also statistically significant (*p* < 0.001). The mean and standard deviation of the water contact angle values were observed for distinct conditions: control (109.78°±6.51), PT (45.28°±5.19), and PT + ZnO (92.68°±7.90).

### Tensile bond strength

**Fig. 5:**
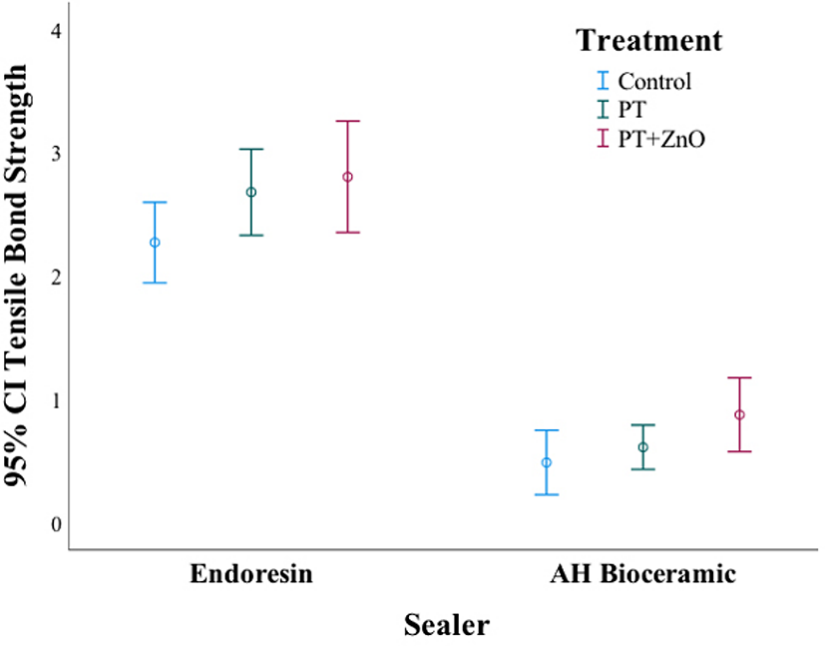
Mean tensile bond strength values (MPa, *n* = 10) of untreated (control), treated with Ar plasma (PT), and functionalized gutta-percha surfaces (plasma treatment followed by ZnO thin film deposition – PT + ZnO) to endodontic sealers

Figure 5 shows the mean tensile bond strength values (MPa) of untreated (control), treated with Ar plasma (PT), and functionalized (PT + ZnO) GP surfaces. Both sealers had some degree of adhesiveness to non-treated GP (control) but with a significant difference between them (F(1,54) = 274.7; *p* < 0.001). The mean bond strength value in Endoresin group was significantly higher than that in AH Plus bioceramic group, for all GP conditions (control, PT and PT + ZnO). There were significant differences concerning treatments (*p* = 0.008) (Table 1).

**Table 1:** ANOVA of tensile bond strength (df: degrees of Freedom, Sig: P value)

Source	Sum of type III squares	f	Mean square	F	Sig.
Sealer	55.738	1	55.738	274.691	< 0.001
Treatment	2.133	2	1.066	5.255	0.008
Sealer * Treatment	0.203	2	0.102	0.500	0.609
Error	10.957	54	0.203		
Total	69.032	59			

Dependent Variable: Tensile Bond strength

a. R Squared = 0.841 (Adjusted R Squared = 0.827)

**Fig. 6:**
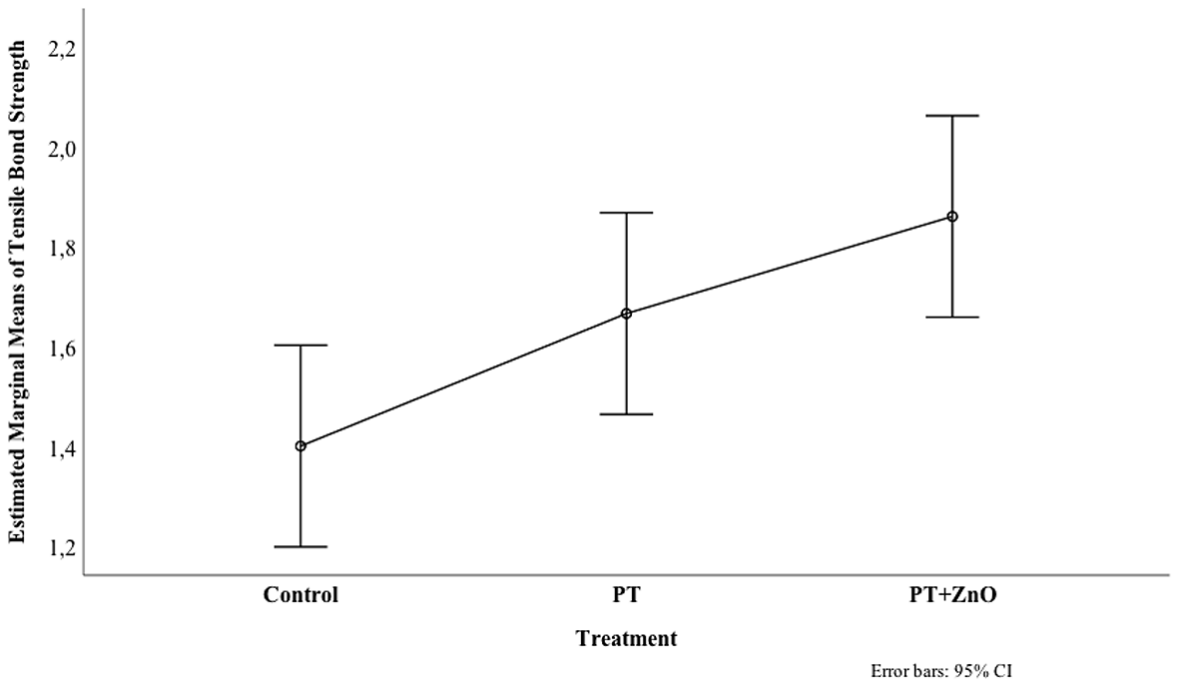
Comparation of estimated marginal means for tensile bond strength of untreated (control), treated with Ar plasma (PT), and functionalized gutta-percha surfaces (plasma treatment followed by ZnO thin film deposition – PT + ZnO) (Bonferroni multiple comparisons test)

Bonferroni multiple comparisons test (Fig. 6) showed a statistically significant difference between the mean bond strength values of the control and the functionalized (PT + ZnO thin film) GP (*p* = 0.006), with the latter presenting the highest bond strength value. There was no significant difference in bond strength values between PT and PT + ZnO (*p* = 0.553), nor between PT and control (*p* = 0.203).

## Discussion

Achieving a tridimensional sealing of the root canal system while preventing coronal and apical leakage is crucial for the root canal treatment outcome [29]. Despite the great development in endodontic materials, studies still show the presence of interfacial gaps in root canal fillings, independent of sealers’ chemical composition, GP type or filling techniques [16, 30, 31]. Minimizing gaps is clinically relevant, preventing bacteria and their by-products to colonize and degrade filling materials. On the other hand, the use of filling materials with improved antibacterial properties is recommended to prevent microbial re-infection [14].

The present investigation evaluated the impact of the GP surfaces modified by plasma treatment and functionalized with ZnO thin film deposition on its adhesiveness to sealers with different chemical compositions: epoxy resin-based (Endoresin) and calcium silicate-based (AH Plus Bioceramic). PT was used to etch, clean, and activate the GP surfaces resulting in the exposure of the ZnO crystals and simultaneously enhancing the adhesion of the ZnO thin film [14, 27]. GP surfaces’ morphology, water contact angle and tensile bond strength were analyzed by comparing distinct GP samples: non-treated (control), treated with Ar plasma (PT), and functionalized by depositing a ZnO thin film (PT + ZnO). The main findings to be stressed are that: (i) the epoxy resin-based sealer (Endoresin) showed a statistically higher mean bond strength value than AH Plus Bioceramic, in all conditions studied, (ii) functionalized GP (PT + ZnO) presented an increased hydrophilicity, compared to the control (not treated GP), and (iii) functionalized GP (PT + ZnO) seems to improve sealer’s adhesion. The present sample data was inconsistent with the null hypothesis, stating that GP surface’s functionalization will not have impact on sealer adhesion. So, the null hypothesis was rejected. With the limitations of the present study, the alternative hypothesis should be considered, i.e., the deposition of a ZnO thin film on GP surface might increase GP’s adhesion to filling materials such as Endoresin and AH Plus Bioceramic sealers.

To evaluate the wettability, the water contact angle was measured on the distinct GP surfaces (untreated and modified). The water contact angle serves as a measure of how a liquid interacts with a solid surface [32]. A lower contact angle implies that the liquid spreads more on the surface, indicating better wettability [32]. This wettability is directly associated with an increase in the surface free energy, leading to a higher affinity for establishing new bonds, which typically results in improved adhesion [32]. The results revealed that the untreated GP is hydrophobic (mean water contact angle of 109.78°), while the modified GP exhibits significantly improved wettability. Yet, it was observed that the mean water contact angle is lower for the GP treated with PT (45.28°) compared to the GP functionalized with the ZnO thin film (92.68°). The observed difference is primarily attributed to the exposure of ZnO crystals, following the treatment into the Ar plasma atmosphere. The ZnO thin film deposition may have masked the surface characteristics induced by PT, increasing the water contact angle of functionalized GP (PT + ZnO) compared to PT.

PT can induce several significant phenomena, including: (i) eliminating superficial contaminants (cleaning); (ii) altering surface morphology and topography through etching; (iii) activating the surface by generating new reactive species, resulting in the formation of novel chemical groups, crosslinking, and chain scission [25]. GP comprises an organic polymeric matrix (GP polymer, resins, and waxes) with embedded inorganic components (zinc oxide and barium sulfate) [11]. Our findings confirm that the PT, promoted into an Ar atmosphere, with the selected parameters (50 W; 60s) [27] was responsible for the remotion of the organic layer on the GP surface (Fig. 3). The PT effectively promoted the surface etching (cleaning superficial contaminants) and activation, exposing the ZnO crystals and increasing the surface’s porosity [15, 27]. These effects result in the creation of new chemical anchoring points, which may favor adhesion. Additionally, the deposition of the ZnO thin film on Ar pre-treated GP, reproduced the main features of the GP surface topography, covering the whole surface. Moreover, the addition of ZnO thin films alters the microstructure of the GP (Fig. 3). The reduced size of the ZnO particles, compared to the PT treatment, would also contribute to an improved adhesion. Smaller particles possess a larger surface area relative to their volume, providing more opportunities for bonding and creating a more interlocked or mechanically entangled structure that enhances adhesion. A previous study [14] stressed the homogeneous layer of ZnO thin film deposited on Ar plasma treated GP surfaces. Furthermore, the significant enhancement on the antimicrobial/antibiofilm ability of ZnO deposition after PT, compared to the film deposited on the native organic layer of the control (not treated GP) has been highlighted. This coating seems to be responsible for reducing the surface porosity in comparison with untreated PT cones. Contrarily, the immersion of GP cones in sodium hypochlorite caused significant surface irregularity which might favor biofilm adhesion [14].

In the last few years, several approaches have been proposed to enhance the adhesive characteristics of GP [11, 15, 16, 27]. The use of coated GP cones incorporated with bioceramic nanoparticles have been suggested, with contradictory findings. Eltair et al. [16] through SEM analysis, concluded that the interface between bioceramic GP cones and CSS was not satisfactory, independently of the obturation technique (lateral compaction or single cone). Bankantan et al. [33] did not find a superior shear bond strength of the CSS. Recently, Quaresma et al. [31] reported that the push-out bond strength between obturated teeth with CSS sealers and bioceramic GP cones showed higher bond strength values, compared to conventional GP and epoxy resin-based sealer. However, different methodologies can influence the bond strength analysis. In the present investigation, Endoresin displayed a stronger bond strength to conventional (non-treated) GP samples than the calcium silicate-based sealer AH Plus Bioceramic, corroborating other studies [31, 34, 35], which indicate varying adhesion levels with sealers of different chemical compositions. Furthermore, it should be noted that bond strength of both sealers was substantially enhanced after functionalization of GP surfaces (PT + ZnO thin film deposition), compared to the control.

There are few studies exploring the sealer adhesion ability to GP [3, 34–36]. Shear, tensile and push-out bond strength tests have been used and contradictory results have been stated due the heterogeneity of the experimental methodologies [3, 34, 36, 37]. As limitations of the current study, it is important to highlight that commercially available GP may vary in its composition depending on the manufacturer. It is conceivable that variations in bond strength values may occur if an alternative brand of GP is used as the substrate or a different assessment methodology is considered. In addition, the tensile bond strength test is a sensitive technique because small changes in the sample or in stress distribution during the load application can affect the results [39]. Further studies with different brands of gutta-percha and sealers are needed to confirm the clinical relevance of the present findings.

## Conclusion

The present findings suggest that the functionalization of GP with a nanostructured ZnO thin film favors the bond strength ability with sealers of different chemical composition. The ZnO thin film deposition preserved GP’s surface morphology modified by PT, enhancing the bond strength to both Endoresin and AH Plus Bioceramic sealers, compared to untreated GP. Further investigation is required to obtain enough scientific evidence to suggest it as a valid alternative to the conventional GP core filling material in root canal treatment.

## Data Availability

The datasets used and/or analysed during the current study available from the corresponding author on reasonable request.
